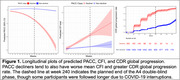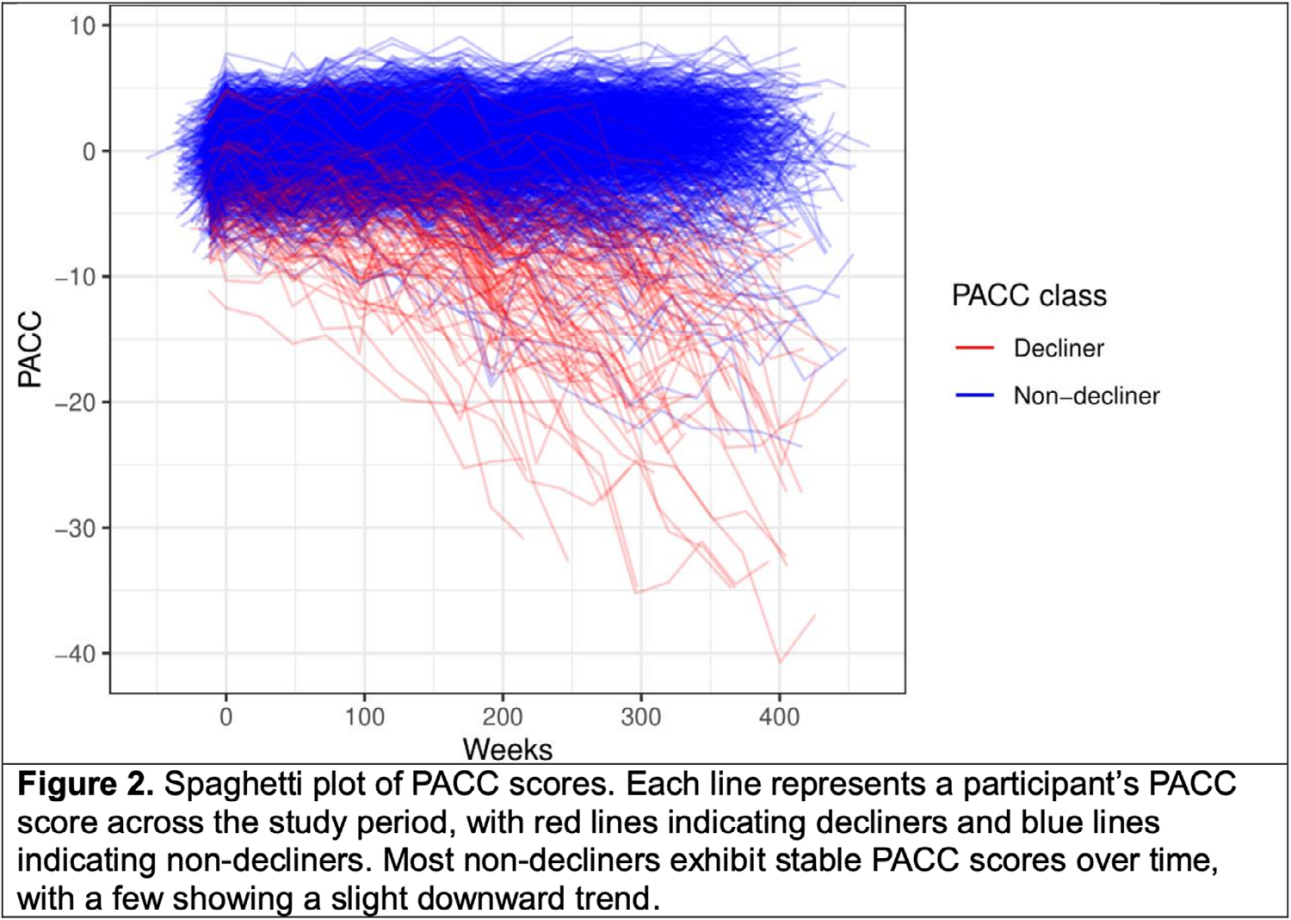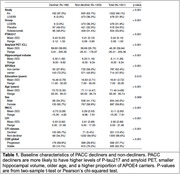# Divergent latent classes of cognitive decline in the A4 and LEARN studies

**DOI:** 10.1002/alz70861_108966

**Published:** 2025-12-23

**Authors:** Runpeng Li, Oliver Langford, Philip S. Insel, Reisa A. Sperling, Paul S. Aisen, Rema Raman, Michael C. Donohue

**Affiliations:** ^1^ Alzheimer's Therapeutic Research Institute, University of Southern California, San Diego, CA USA; ^2^ University of California, San Francisco, San Francisco, CA USA; ^3^ Center for Alzheimer Research and Treatment, Brigham and Women’s Hospital, Massachusetts General Hospital, Harvard Medical School, Boston, MA USA

## Abstract

**Background:**

Recent findings from A4, a randomized trial of solanezumab for preclinical Alzheimer’s Disease, and LEARN, an observational study of amyloid negative individuals, suggest that Alzheimer’s biomarkers in cognitively healthy adults are linked to cognitive and clinical progression. Specifically, higher baseline amyloid PET CL and *p* ‐tau217 levels are associated with greater PACC decline. However, there is considerable heterogeneity in this decline.

**Method:**

We apply a Latent Class Mixed‐Effects Model (LCMM), an approach which combines latent class analysis with mixed effects modeling, to data from both studies to characterize and explore predictors of this heterogeneity. We examine the value of baseline plasma *p* ‐tau217, amyloid PET, sex, age, education, hippocampal volume, and treatment with solanezumab for predicting class membership and PACC change. Longitudinal trends are modeled with natural cubic splines with two degrees of freedom, and participant‐specific random intercepts and slopes. Bayesian Information Criterion (BIC) and Integrated Completed Likelihood (ICL) are used to identify the optimal number of classes.

**Result:**

We identify two latent classes (non‐decliners and decliners) of PACC progression in A4 and LEARN with 81.2% of A4 participants and 98.7% of LEARN participants predicted to belong to the cognitively stable class, namely the non‐decliners. Table 1 details baseline characteristics by latent class. Decliners had a greater proportion of *APOE* ε4 carriers, and greater mean age, amyloid PET CL values, *p* ‐tau217 values, and hippocampal atrophy. In the LCMM, *p* ‐tau217 levels (*p* <0.001), amyloid PET CL values (*p* <0.001), and hippocampal volume (*p* =0.007) were significant predictors of latent class, while sex and education were not. Additionally, PACC decliners showed worse mean CFI scores over time, and greater CDR global progression (Figure 1). Figure 2 demonstrates the individual PACC trajectories colored by predicted latent classes.

**Conclusion:**

The analysis shows striking divergent classes, with *p* ‐tau217 levels and amyloid PET CL values emerging as strong group‐level predictors of class membership. However, neither biomarker is an accurate individual‐level classifier of latent class. This analysis suggests that more sensitive cognitive measures or longer follow‐up will be needed to detect a therapeutic benefit on cognition for most individuals, and supportive biomarker evidence will be essential for regulatory approval.